# Establishing Renal Proximal Tubule Epithelial-Derived Cell Lines Expressing Human Telomerase Reverse Transcriptase for Studying BK Polyomavirus

**DOI:** 10.1128/MRA.01129-19

**Published:** 2019-10-17

**Authors:** Linbo Zhao, Michael J. Imperiale

**Affiliations:** a Department of Microbiology and Immunology, University of Michigan, Ann Arbor, Michigan, USA; b Comprehensive Cancer Center, University of Michigan, Ann Arbor, Michigan, USA; Indiana University, Bloomington

## Abstract

We previously established an infection model for BK polyomavirus (BKPyV) in primary human renal proximal tubule epithelial (RPTE) cells. Use of these cells is limited by their inability to be passaged extensively. Here, we describe RPTE cells immortalized with human telomerase reverse transcriptase (hTERT), which can serve as a model system for acute or persistent BKPyV infection.

## ANNOUNCEMENT

BK polyomavirus (BKPyV) was first isolated in 1971 (1). BKPyV is a member of the Polyomaviridae, which is a group of small, icosahedral, nonenveloped viruses with circular double-stranded DNA genomes. Several systems for studying BKPyV-rearranged variants, which are generally isolated from patients with BKPyV disease and can replicate robustly in culture, have been established (2, 3). Our lab previously demonstrated that primary human renal proximal tubule epithelial (RPTE) cells could serve as an acute lytic infection model for studying the BKPyV Dunlop variant, and other variants, *in vitro* (4). One of the disadvantages of primary RPTE cells is that they divide slowly and stop growing at around passage 10. To extend the time window for BKPyV research, we generated human telomerase reverse transcriptase (hTERT)-expressing RPTE (RPTE-hTERT) cells.

**FIG 1:**
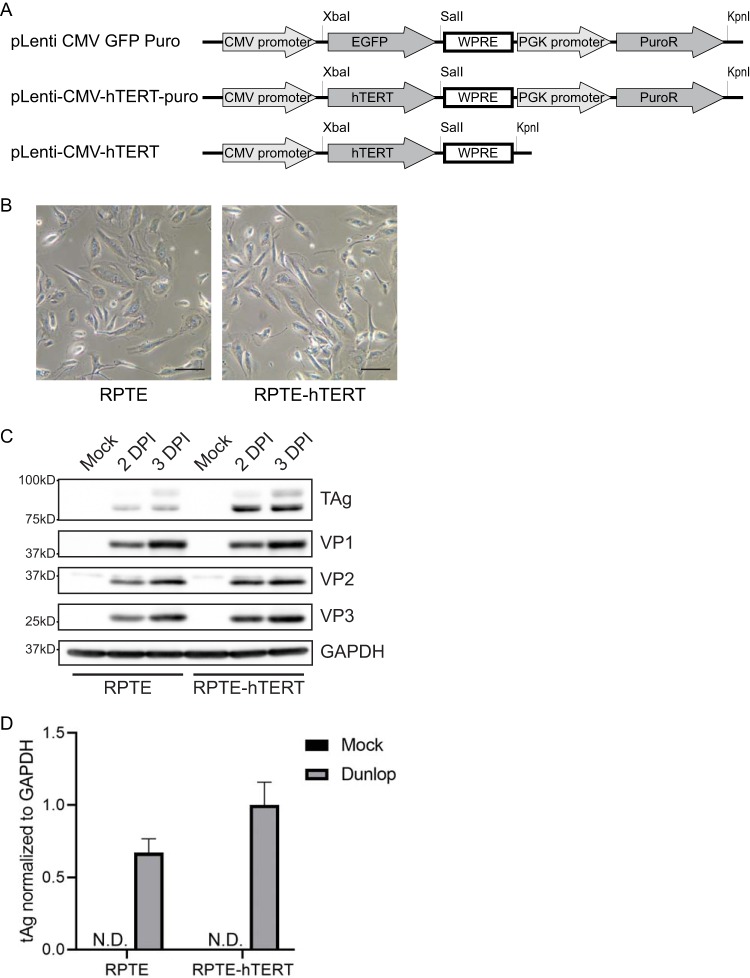
(A) Schematic diagram of the lentivirus plasmid construction. (B) Phase-contrast images of RPTE and RPTE-hTERT cells. RPTE cells were plated 1 day before taking images. Images were taken using a phase-contrast microscope. Bar, 100 μm. (C) Viral protein expression in RPTE and RPTE-hTERT cells. RPTE or RPTE-hTERT cells were infected by BKPyV at a multiplicity of infection (MOI) of 1. Viral early protein large tumor antigen (TAg), late proteins VP1, VP2, and VP3, and glyceraldehyde-3-phosphate dehydrogenase (GAPDH) were examined by Western blotting. (D) Viral early protein small tAg expression in RPTE and RPTE-hTERT cells. RPTE or RPTE-hTERT cells were infected by BKPyV at an MOI of 1. tAg mRNA expression was examined by reverse transcription-quantitative PCR (RT-qPCR). N.D., not detected.

RPTE cells were acquired from Lonza and grown according to our previous report (5). RPTE and RPTE-hTERT cells were maintained in renal epithelial cell growth medium kit (REGM/REBM; Lonza). An earlier publication showed that hTERT can be used to immortalize RPTE cells (6). However, the lentivirus vector (pLXSN) used in the previous report contains an SV40 origin of replication, which could lead to problems due to its ability to replicate in the presence of BKPyV. We therefore started with another lentivirus vector to avoid the use of SV40 sequences. pLenti CMV GFP Puro (658-5) was a gift from Eric Campeau and Paul Kaufman (plasmid number 17448; Addgene) (7). hTERT was amplified with a primer pair (XbaI-Kozak-hTERT-F, 5′-AAATCTAGAGCCGCCACCATGCCGCGCGCTCCCCGCTGC-3′, and SalI-hTERT-R, 5′-AGGGTCGACTCAGTCCAGGATGGTCTTGAA-3′). We first prepared an intermediate plasmid (pLenti-CMV-hTERT-puro) by substituting GFP in the pLenti CMV GFP Puro plasmid with hTERT at the XbaI and SalI sites (Fig. 1A). In the second step, the woodchuck hepatitis virus posttranscriptional regulatory element (WPRE) sequence was amplified with a primer pair (SalI-WPRE-F, 5′-TGAGTCGACAATCAACCTCTGGAT-3′, and KpnI-WPRE-R, 5′-AAAGGTACCAGGCGGGGAGGCGGCCCAA-3′), and the puromycin selection markers in pLenti-CMV-hTERT-puro were deleted to construct the final pLenti-CMV-hTERT plasmid by substituting the fragment between KpnI and SalI sites with the amplified WPRE. The hTERT-WPRE region was sequenced to confirm the integrity of PCR amplification and cloning.

hTERT-expressing lentivirus was produced by cotransfecting the pLenti-CMV-hTERT, pRSV-Rev, pMDLg/pRRE, and pMD2.G plasmids into 293TT cells (8, 9). Fresh medium was supplied 16 h posttransfection. Medium containing lentivirus was harvested at 48 and 72 h posttransfection and filtered with a 0.45-μm polyethersulfone filter (MilliporeSigma). Filtered medium was overlaid on 20% sucrose in 1× phosphate-buffered saline, and lentivirus was concentrated by centrifuging at 24,000 rpm for 2 h (AH-629 rotor). Pelleted lentiviruses were resuspended in complete REGM/REBM.

RPTE cells at passage 3 were grown in REGM/REBM medium in a 10-cm dish. hTERT-expressing lentivirus at a multiplicity of infection (MOI) of 0.3 was directly added to the cells and inoculated at 37°C overnight. Cells were passaged 3 days postransduction and further passaged at 70% confluence until passage 20 to select against nontransduced cells. Single RPTE-hTERT subclones were isolated by seeding hTERT-transduced cells at passage 20 in 96-well plates at a concentration of 0.2 cells per well. Subclones were subsequently expanded in 6-well plates and 10-cm dishes before freezing down aliquots in REBM/REGM with 10% dimethyl sulfoxide (DMSO) and 10% fetal bovine serum (FBS) in liquid nitrogen. hTERT integration was confirmed by amplifying hTERT-WPRE fragment from cellular genomic DNA and Sanger sequencing (data not shown). Images of RPTE-hTERT cells are shown in Fig. 1B.

To test if RPTE-hTERT cells are susceptible to BKPyV infection, RPTE-hTERT cells and RPTE cells were infected with BKPyV (Dunlop) at an MOI of 1, as previous described (10). Protein samples were harvested with E1A buffer (50 mM HEPES [pH 7], 250 mM NaCl, and 0.1% NP-40, with the following inhibitors added right before use: 5 μg/ml phenylmethylsulfonyl fluoride [PMSF], 5 μg/ml aprotinin, 5 μg/ml leupeptin, 50 mM sodium fluoride, and 0.2 mM sodium orthovanadate). Equal amounts of protein were electrophoresed on a 4 to 15% precast protein gel (Bio-Rad). The separated proteins were transferred to nitrocellulose membranes with the Trans-Blot Turbo transfer system (Bio-Rad). Membranes were blocked in 2% nonfat milk in PBS-Tween (PBS-T) buffer (144 mg/liter KH_2_PO_4_, 9 g/liter NaCl, 795 mg/liter Na_2_HPO_4_ [pH 7.4], and 0.1% Tween 20) for 1 h at room temperature. Antibodies for Western blotting were diluted in 2% milk in PBS-T as follows: anti-large tumor antigen (TAg) mouse ascites (pAb416) at 1:5,000 (11); anti-glyceraldehyde-3-phosphate dehydrogenase (GAPDH; MilliporeSigma, CB1001) at 1:20,000; anti-VP1 (pAb5G6) mouse ascites at 1:5,000; custom-made rabbit anti-VP2 (Bethyl Labs) at 1:10,000 (12); horseradish peroxidase (HRP)-conjugated enhanced chemiluminescence (ECL) sheep anti-mouse (NA931V; GE Healthcare) at 1:5,000; and HRP-conjugated ECL donkey anti-rabbit antibody (NA934V; GE Healthcare) at 1:5,000. The probed membrane was developed with HRP substrate (WBLUF0100; MilliporeSigma), and images were acquired with the Syngene PXi gel doc system. Western blotting showed that there was a slight increase of viral early protein large tAg at 48 h postinfection in the RPTE-hTERT cells compared to the that in the RPTE cells (Fig. 1C), while late protein VP1, VP2, and VP3 expression was similar between RPTE and RPTE-hTERT cells, which suggested an accelerated early phase of the virus life cycle because the RPTE-hTERT cells were actively dividing. Due to a lack of small tAg antibody, tAg expression was examined by reverse transcription-quantitative PCR (RT-qPCR). Total cellular RNA was harvested with TRIzol RNA isolation reagent (Thermo Fisher Scientific) and purified with a spin column-based purification kit (Zymo Research). cDNA was synthesized with SuperScript III reverse transcriptase (Thermo Fisher Scientific). qPCR was performed on the cDNA with PowerUp SYBR qPCR mastermix (Thermo Fisher Scientific) and the following primers for each target, as follows: GAPDH (5′-GCCTCAAGATCATCAGCAAT-3′ and 5′-CTGTGGTCATGAGTCCTTCC-3′) and small tumor antigen (5′-CAGTGCACAGAAGGCTTTTTGGAACA-3′ and 5′-AGCCTGATTTTGGAACCTGGAGTAGC-3′). The qRT-PCR results measuring the mRNA are concordant with the previously observed increase in protein production as identified by Western blotting 48 h postinfection in the immortalized cells (Fig. 1D).

We believe that the RPTE-hTERT cell line will be a useful tool for studying biological aspects of BKPyV infection that cannot be performed in primary cells with limited life spans in culture.

### Data availability.

RPTE-hTERT cells are provided upon request by contacting the corresponding author.
